# Saccade metrics reflect decision-making dynamics during urgent choices

**DOI:** 10.1038/s41467-018-05319-w

**Published:** 2018-07-25

**Authors:** Joshua A. Seideman, Terrence R. Stanford, Emilio Salinas

**Affiliations:** Department of Neurobiology and Anatomy, Wake Forest School of Medicine, 1 Medical Center Blvd., Winston-Salem, NC 27157-1010 USA

## Abstract

A perceptual judgment is typically characterized by constructing psychometric and chronometric functions, i.e., by mapping the accuracies and reaction times of motor choices as functions of a sensory stimulus feature dimension. Here, we show that various saccade metrics (e.g., peak velocity) are similarly modulated as functions of sensory cue viewing time during performance of an urgent-decision task. Each of the newly discovered functions reveals the dynamics of the perceptual evaluation process inherent to the underlying judgment. Remarkably, saccade peak velocity correlates with statistical decision confidence, suggesting that saccade kinematics reflect the degree of certainty with which an urgent perceptual decision is made. The data were explained by a race-to-threshold model that also replicates standard performance measures and cortical oculomotor neuronal activity in the task. The results indicate that, although largely stereotyped, saccade metrics carry subtle but reliable traces of the underlying cognitive processes that give rise to each oculomotor choice.

## Abstract (web summary)

Saccades have been extensively used to report choices in perceptual decision making studies yet little is known about the influence of covert decision-related processes on saccade metrics. Here, the authors demonstrate that saccade kinematics is a reliable tell about the degree of decision certainty.

## Introduction

The empirical study of perceptual decision making hinges on the ability to make inferences about covert cognitive states based on overt behaviors. And yet, while saccadic choice paradigms have been instrumental in advancing our understanding of decision making in general, few studies have directly linked saccade metrics themselves to underlying decision-related processes. Thus, it is currently unknown whether and how the formation and development of a perceptual decision influence the metrics of a saccadic choice upon execution, or complementarily, whether the metrics of saccades are a reliable tell of perceptual decision-making dynamics.

Physiologically, decision-related processing as well as saccadic motor planning and execution are known to have at least partly overlapping neural substrates. Within oculomotor regions, such as the frontal eye field (FEF) and superior colliculus (SC), putative perceptual decision variables have been shown to be encoded within the firing rates of neurons prior to saccadic choice execution^1–6^. Both empirical as well as theoretical results indicate that when firing rates within these brain regions reach a certain threshold of activation, a saccade is triggered^5,7–9^. While the amplitude and direction of the movements that ensue are primarily encoded by the locus of neural activity^10–14^, there is also evidence that saccadic peak velocity (independent from amplitude) is influenced by the overall level and/or temporal pattern of activation within these motor maps^15–20^. Taken together, these studies implicate oculomotor areas in a sensorimotor transformation whereby perceptually-driven changes in activity influence saccade kinematics. However, as noted above, direct behavioral evidence in support of this hypothesis is lacking, and there is essentially no mechanistic understanding of the process by which this could occur.

Therefore, we sought to determine if, how, and when perceptual decision-making dynamics influence the metrics of saccadic eye movements. We investigate this using a recently developed, urgent saccadic choice task in which perceptual performance depends on processing time, i.e., sensory cue viewing time prior to saccade onset^5,6,21,22^. Within this urgent paradigm, accurate performance requires coordinated, dynamic interaction between perceptual and motor systems in the moments leading up to saccade execution — facilitating, as evidenced by the data presented in this paper, overt manifestations of a seamless transition from perception to action. Indeed, we find that numerous saccade metrics (e.g., peak velocity, endpoint scatter, etc.) vary continuously as functions of processing time and that changes in these metrics closely coincide with processing-time-dependent changes in perceptual choice accuracy. In addition, using a race-to-threshold model previously proven to replicate standard performance metrics in the task (e.g., choice accuracy, response time), as well as simultaneously recorded FEF neuronal activity^5,6,21,22^, we provide a plausible physiological mechanism by which perceptually-driven changes in oculomotor firing rates could influence the peak velocity of saccadic eye movements. Ultimately, our empirical and theoretical lines of evidence converge to support a unified, mechanistic framework, whereby sensory evidence informs not only what saccadic choices we make, but when and how we make them.

## Results

### Urgent perceptual discriminability is a matter of time

**Fig. 1:**
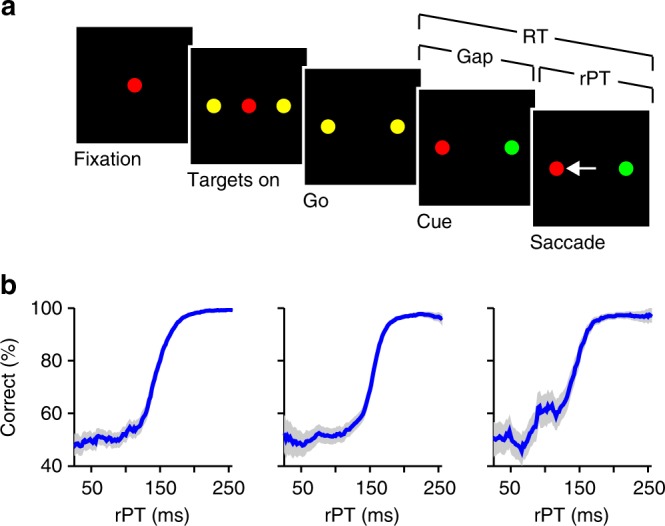
CS task and psychophysical performance of three monkeys. **a** The CS task. The color of the initial fixation spot (Fixation) defines the color of the eventual target. Two identical yellow spots, diametrically opposed, surrounding the central stimulus, represent potential target locations (Targets on/Go). Disappearance of the central fixation spot represents the command to move, or go signal (Go). Target and distracter are revealed (Cue) only after a variable gap of time (Gap) following the go signal. Choices are indicated via saccadic eye movement (white arrow). Reaction time (RT) is measured between the onset of the go signal and the onset of the saccadic response. Raw processing time (rPT) is measured between cue onset and saccade onset. **b** Percentage of correct responses as a function of rPT (tachometric curves). Error bars in **b** represent 95% binomial proportion confidence intervals. From left to right, data are from monkeys R (*n* = 19,796 trials), T (*n* = 11,148 trials), and G (*n* = 6042 trials)

In the current study, three monkeys performed the compelled-saccade (CS) task — an urgent, top-down search task that systematically varies the amount of time available to perceptually evaluate sensory cue information before committing to a saccadic choice (Fig. 1a). Unlike traditional saccadic choice tasks, the CS task presents the go signal before revealing target and distracter. Consequently, saccadic motor planning starts first, and it is only later, after an unpredictable period of time (gap; 25–250 ms), that the sensory cues to be discriminated (two colored spots) are presented (cue), and perceptual information can guide the already ongoing saccadic choice process.

Perceptual performance in the task fundamentally depends on the raw processing time (rPT), which is the amount of time available to view the cue information prior to saccade onset (in each trial, rPT = RT − gap; Fig. 1a). This is evident by plotting choice accuracy versus rPT to produce a perceptual performance measure that we refer to as the “tachometric curve” (Fig. 1b). The tachometric curves from all three subjects reveal that, as a function of rPT, saccadic choices range from uninformed guesses (performance is near chance at rPTs < 100 ms) to informed discriminations (performance is >95% correct at rPTs > 200 ms), with perceptual information modulating choice accuracy at an extremely rapid rate starting ~125 ms after cue onset (i.e., ~125 ms rPT; Fig. 1b). These data demonstrate that, in the CS task, saccades are executed at various points throughout the temporal evolution of a perceptual decision. In the sections that follow, we examine the degree to which the state of the perceptual decision-making process at the time of saccade commitment influences the kinematics of the ensuing eye movement.

### Perceptual information modulates saccadic peak velocity

**Fig. 2:**
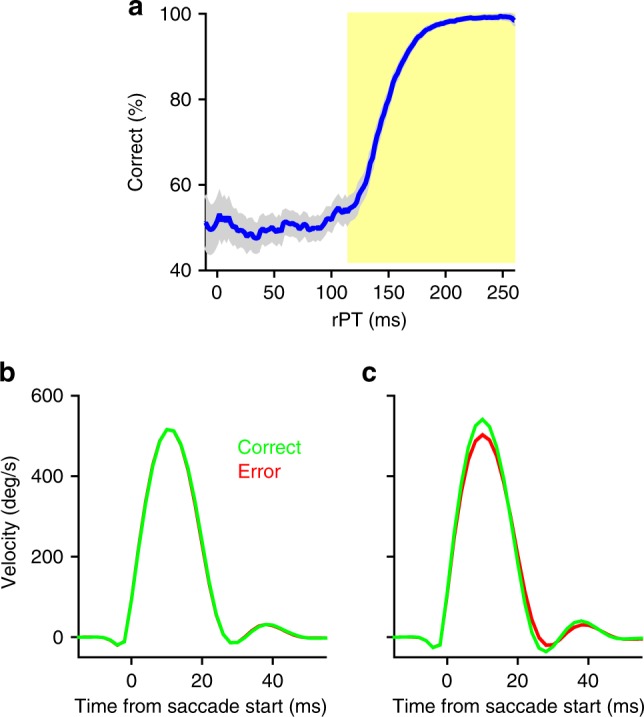
Eye velocity profiles show evidence of a perceptual influence on saccade kinematics. **a** Tachometric curve (blue). Trials were divided into two rPT ranges — short (white region) and long (yellow region) — according to the tachometric curve. Error bars represent 95% binomial proportion confidence intervals. **b**, **c** Mean eye velocity (±1 standard error of the mean; s.e.m.) of correct (green) and incorrect (red) saccadic choices executed at short (**b**) and long (**c**) rPTs. Velocity traces are plotted as functions of time from saccade onset. Data are from monkey R

Using the CS task, we set out to determine what relationship, if any, exists between the velocity of a saccade and the temporal availability of sensory information that is relevant for guiding a perceptual decision. Toward this end, we divided saccadic responses by processing time (short and long, according to the tachometric curve; Fig. 2a) and choice outcome (correct and incorrect), and compared their velocity profiles. At short rPTs, the average velocity profiles of correct and incorrect saccades were indistinguishable — consistent with the idea that these choices (guesses) were not guided by the cue information (Fig. 2b). In contrast, subtle but highly significant differences were evident between the velocity profiles of correct and incorrect saccades at long rPTs (mean peak velocity, correct versus incorrect: monkey R, *p* = 10^−37^; monkey T, *p* = 10^−50^; monkey G, *p* = 10^−27^; Wilcoxon rank-sum test; Fig. 2c). Notably, the mean peak velocity of long-rPT correct saccades was slightly (~5%) higher than that of long-rPT incorrect saccades.

**Fig. 3:**
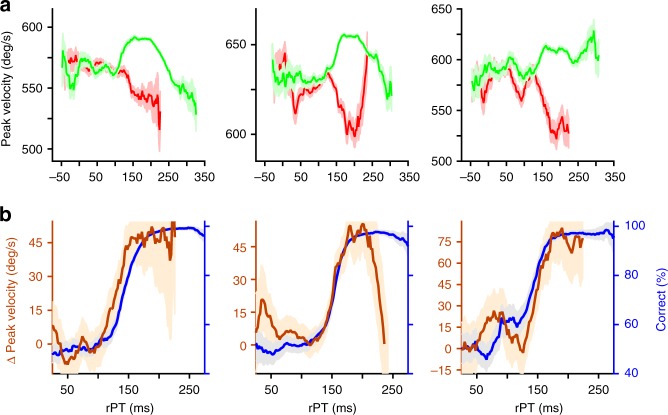
Processing-time-dependent changes in peak velocity closely track perceptual performance. **a** Mean peak velocity (±1 s.e.m.) of correct (green) and incorrect (red) saccadic choices as a function of rPT. **b** Peak velocity modulation (mean correct − mean incorrect peak velocity, brown curves) and choice accuracy (tachometric curves, blue) as functions of rPT. Error bars in **b** represent 95% confidence intervals. Data are from monkeys R, T, and G (left to right)

**Fig. 4:**
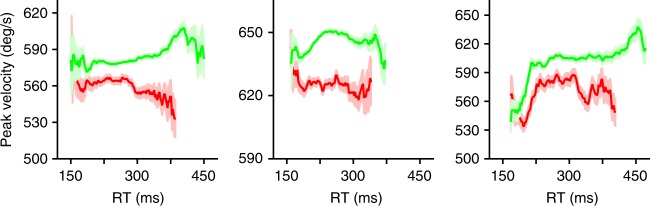
Peak velocity is largely independent from movement preparation time in the CS task. Mean peak velocity (±1 s.e.m.) of correct (green) and incorrect (red) saccadic choices as a function of RT. Data are from monkeys R, T, and G (left to right)

Next, to determine if the changes in peak velocity followed a similar time course to that of the evolving perceptual judgment, we binned responses by rPT and choice outcome and computed peak velocity averages in each bin. The results from each of three subjects show that peak velocity varies continuously as a function of processing time (Fig. 3a). At short processing times (rPTs < 100 ms), peak velocity remained relatively constant and was similar for correct and incorrect choices. Then, after an apparent threshold of exposure to the sensory cue, the peak velocities for correct and incorrect choices diverged abruptly, increasing for the former and decreasing for the latter. Importantly, this split was not simply explicable by differences in movement preparation time, as there was no discernible relationship between peak velocity and RT (Fig. 4). Rather, as Fig. 3a illustrates, the peak velocity of saccadic eye movements in the CS task depends on the amount of time available to process the cue information prior to saccade onset. Although late rPT (rPTs > 225 ms) modulations were also observed, such effects were inconsistent across subjects and appeared to occur only after the tachometric curve had reached its asymptote, i.e., after the perceptual judgment had already completed its development in time. Hereafter, we focus on the earlier (rPTs < 200 ms) bi-directional velocity modulations that were strongly stereotyped in their correlation with choice performance.

To more directly relate the time course of the changes in peak velocity to psychophysical performance, we yoked correct and incorrect velocity averages by taking their difference (correct − incorrect) within processing time bins, rescaled the result, and compared it to the tachometric curve. Plotted together in this way, the similarity in time course is striking, strongly suggesting that perceptual information simultaneously impacts both choice performance and saccade peak velocity (Fig. 3b). Bootstrapping analyses found no significant shift between the velocity and tachometric curves for any of the three subjects, confirming their temporal alignment (difference in alignment: monkey R, *p* = 0.06; monkey T, *p* = 0.53; monkey G, *p* = 0.19; see Methods). In addition, the curves had statistically identical steepness, indicating that perceptual information modulated choice accuracy and saccade velocity at an equivalent rate with respect to rPT (difference in rise time: monkey R, *p* = 0.53; monkey T, *p* = 0.38; monkey G, *p* = 0.41; see Methods). Therefore, in the CS task, cue information speeds up correct saccades toward the target and slows down incorrect saccades toward the distracter, and these effects closely coincide with processing-time-dependent changes in choice performance.

### Perceptual modulation of multiple saccade metrics

**Fig. 5:**
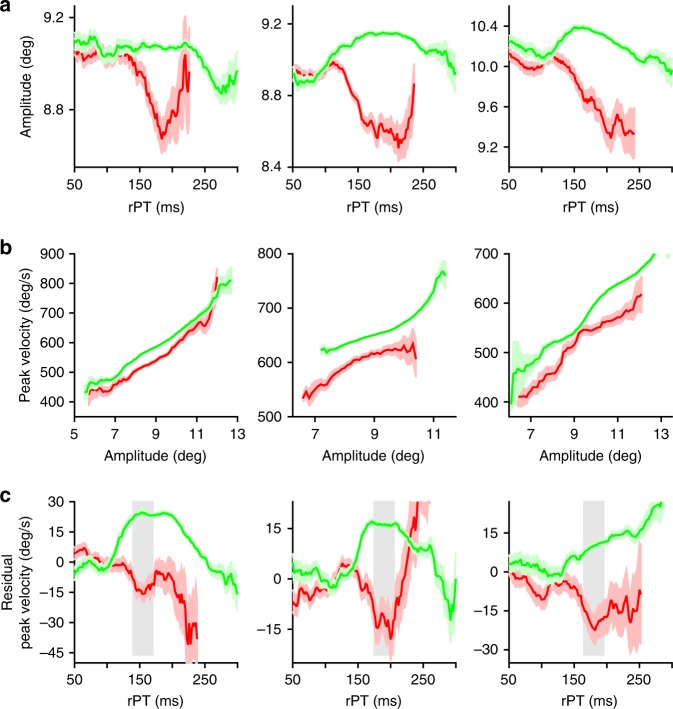
Peak velocity and amplitude are independently modulated by rPT. **a** Mean saccade amplitude (±1 s.e.m.) of correct (green) and incorrect (red) saccadic choices as a function of rPT. **b** Mean peak velocity (±1 s.e.m.) as a function of amplitude (main sequence) of a subset of correct (green) and incorrect (red) long-rPT saccadic choices. **c** Mean residual peak velocity (rpv) ± 1 s.e.m. as a function of rPT. Trials plotted in **b** correspond to those shaded gray in **c**. Data are from monkeys R, T, and G (left to right)

Further analysis revealed that saccade amplitude also depends on processing time (Fig. 5a). That is, with differences on the order of fractions of a degree, correct saccades at long rPTs were hypermetric and incorrect saccades hypometric, on average, when compared to their short-rPT counterparts. Moreover, the pattern of amplitude modulation with rPT resembled that for peak velocity (compare with Fig. 3a). Thus, we wondered whether the observed effects on peak velocity could be explained simply as a direct consequence of the standard association between amplitude and peak velocity, i.e., by the saccadic “main sequence” (ref. ^23^; Fig. 5b). To investigate this, we performed the following analysis. For each saccade, we computed the residual peak velocity (rpv) around the line of best fit between the amplitudes and peak velocities of uninformed saccades (rPTs < 75). This measure of saccade vigor — rpv — quantifies peak velocity enhancement or suppression relative to that of amplitude-matched uninformed trials, such that velocity enhanced saccades have positive rpv and velocity suppressed saccades have negative rpv, still with units of degrees per second (deg/s). Then we plotted mean rpv as a function of processing time. The results revealed processing-time-dependent modulation of saccade vigor for all subjects (Fig. 5c). Specifically, the rpv of correct saccades increased to more positive values, whereas that of incorrect saccades decreased to more negative values as a function of rPT. This confirms that the magnitude of the observed peak velocity effects was beyond that which can be accounted for by changes in saccade amplitude. Together, these results indicate that saccade amplitude and peak velocity are independently modulated according to the amount of time that is available for cue information to guide an urgent perceptual decision.

**Fig. 6:**
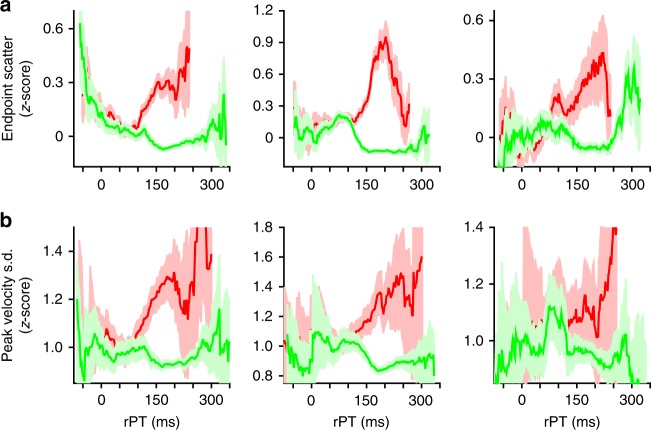
Perceptual information modulates the variability of saccade metrics. **a** Mean saccade endpoint scatter (±1 s.e.m.) of correct (green) and incorrect (red) saccadic choices as a function of rPT. **b** Mean standard deviation (s.d.) of normalized (*z*-scored within each session) peak velocity measures for correct (green) and incorrect (red) saccadic choices as a function of rPT. Error bars in **b** represent 95% confidence intervals. Data are from monkeys R, T, and G (left to right)

Next, we sought to establish whether, during a perceptual decision, incoming cue information influences the variability of the evoked saccades. We first examined the mean endpoint scatter of correct and incorrect saccades for evidence of perceptual modulation (Fig. 6a). At rPTs corresponding to uninformed choices, correct and incorrect endpoint measures did not differ. However, as cue viewing time increased beyond a critical threshold, the endpoints of saccades made to the correct target became less scattered, whereas those of saccades made to the distracter became more scattered. Once again, bootstrapping analyses indicated that the time course of these changes closely matched that of perceptual performance as measured via the tachometric curve (difference in alignment: monkey R, *p* = 0.12; monkey T, *p* = 0.15; monkey G, *p* = 0.85; difference in rise time: monkey R, *p* = 0.49; monkey T, *p* = 0.22; monkey G, *p* = 0.89).

Having discovered this, we hypothesized that variability in the peak velocity of saccades may similarly reflect perceptual processing. Indeed, the standard deviation (s.d.) of the peak velocity depended on rPT in much the same way as the dispersion in saccade endpoint (Fig. 6b). That is, for incorrect choices, the standard deviation of peak velocity sharply increased, while that of correct saccades decreased as a function of rPT. These results clearly demonstrate that the variability of saccade metrics is influenced by the amount of information on which an urgent perceptual judgment is based.

### A plausible mechanism linking perception and peak velocity

Thus far, we have demonstrated that various saccade metrics are highly dependent on the temporal availability of sensory information that is relevant for guiding a perceptual decision. In light of these findings, we sought to determine how (mechanistically) perceptually-driven changes in oculomotor activity could influence saccade metrics. To do so, we utilized a heuristic model that reproduces both behavioral performance and FEF neuronal activity in the CS task — the accelerated race-to-threshold model^5,6,21,22^.

**Fig. 7:**
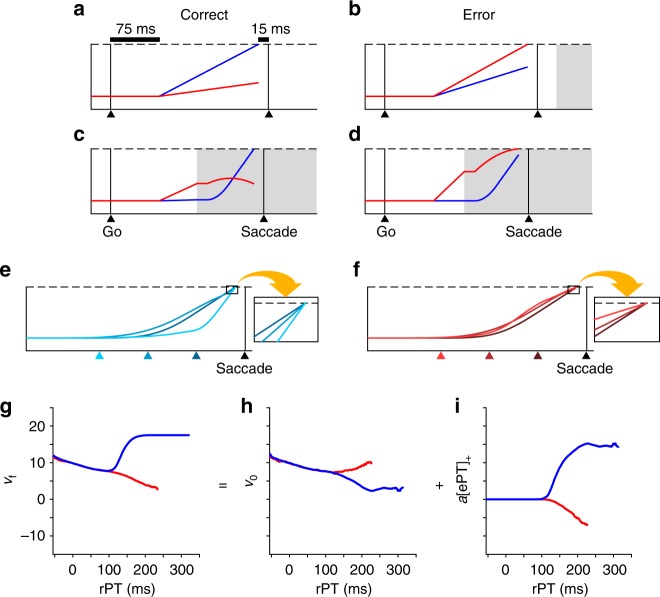
The accelerated race-to-threshold model provides a plausible physiological mechanism by which sensory evidence could influence saccade kinematics. **a**–**d** Four simulated trials of the accelerated race-to-threshold model. In each trial of the model, *x*_L_ (red traces) and *x*_R_ (blue traces) — representing the mean firing rates of two populations of oculomotor neurons — simultaneously rise toward a fixed threshold (dashed lines), competing to trigger a saccade toward the left or right, respectively. For simplicity, we have illustrated only trials in which the target is on the right and the distracter on the left. Black triangles and vertical lines indicate the onset of the go (Go) and saccade (Saccade). Shaded regions represent time after cue arrival. After a short, variable afferent delay following the go signal (fixed here at 75 ms for illustrative purposes only), *x*_L_ and *x*_R_ race toward threshold with initial, constant build-up rates drawn randomly from a bivariate distribution. **a** A correct guess results when the variable congruent with the target (*x*_R_; blue trace) reaches threshold before the competing variable, and before the cue information arrives. **b** An incorrect guess. **c**, **d** Time permitting, the cue information accelerates the variable congruent with the target and decelerates the variable congruent with the distracter. **c** A correct trial in which the motor activity congruent with the target accelerates to threshold after the cue arrives. **d** An incorrect trial in which the variable congruent with the distracter decelerates, but still reaches threshold after the cue arrives. **e**, **f** Mean firing rate trajectories (*x*_R_, *x*_L_) that reached threshold, resulting in correct (**e**) and incorrect (**f**) outcomes at short, intermediate, and long rPTs (dark, intermediate, and light hues, respectively). Colored triangles indicate average times of cue presentation relative to saccade onset (i.e., the time from each colored triangle until saccade is equal to the mean rPT of the simulated trials in that bin). Responses are aligned on saccade onset. **g**–**i** Mean *v*_f_ (**g**), *v*_0_ (**h**), and *a*[ePT]_+_ (**i**) of correct (blue) and incorrect (red) trials as functions of rPT. Simulations were based on the behavioral data from subject R

In the model, saccadic choices are contingent on the outcome of a race to threshold between two variables, *x*_L _and *x*_R_, which represent the mean firing rates of two populations of oculomotor neurons, each competing to initiate an eye movement to one of the two potential target locations. In each simulated trial, the go signal triggers both motor plans to race toward threshold, with initial, constant build-up rates $\left( {v_0^{\mathrm{L}},v_0^{\mathrm{R}}} \right)$ drawn randomly from a bivariate distribution. Next, time permitting, incoming cue information simultaneously accelerates the plan congruent with the target (acceleration equals *a*_T_, which is positive) and decelerates the plan congruent with the distracter (acceleration equals *a*_D_, which is negative). In this way, the model simulates both correct (Fig. 7a, c) and incorrect responses (Fig. 7b, d) that may correspond to either guesses (Fig. 7a, b) or informed choices (Fig. 7c, d), depending on the timing of the cue relative to how advanced are the motor plans toward the target (blue traces) and distracter (red traces).

As can be seen in Fig. 7e, f, the simulated motor plan trajectories that give rise to correct and incorrect saccadic choices vary, on average, depending on rPT. As our current interest relates to saccade execution specifically, we examined the state of the simulated motor plans around the time of saccade initiation as a function of rPT (Fig. 7e, f insets) in search of processing-time-dependent modulation relating to that of saccade metrics. Indeed, using the accelerated race-to-threshold model — fit to reaction time data (see model fits, Supplementary Fig. 1; see Methods) and blind to all saccade metrics — we found that, as a function of processing time, the derivative of the simulated firing rates at threshold crossing (*v*_f_) exhibited a pattern of modulation remarkably similar to that of peak velocity (Fig. 7g; compare with Fig. 3a). That is, on average, the *v*_f_ values from correct and incorrect simulated trials were virtually identical up to a point (around rPT = 125 ms) and then split, with those from correct saccades increasing and those from incorrect saccades decreasing thereafter (given only slight adjustments, the model can account for additional, late rPT peak velocity modulation as well; see Methods). Importantly, the time course of these simulated results was statistically similar to that of the observed peak velocity effects (difference in alignment: *p* = 0.12; difference in rise time: *p* = 0.25). Together, these simulations provide a plausible mechanistic explanation for our saccade metrics data, demonstrating how perceptual decision-making dynamics could influence saccade peak velocity via rate coding within the FEF. Notably, other simulated quantities calculated just prior to saccade onset (e.g., the difference between in vs. away activity, the activity integrated over a given time window, etc.) did not behave in the same way as *v*_f_; i.e., they showed trends as functions of rPT that differed from those seen in the peak velocity data. Thus, the model specifically suggests that the derivative of the firing rate at threshold crossing in a given trial is closely related to the measured peak saccade velocity.

Further insight into the origin of this effect can be gleaned from the model. As previously described, saccadic choices depend not only on when the cue information arrives, but also on the state of the already developing motor plan at that time (corresponding to a random, initial guess driven by urgency). On each simulated trial, the derivative of the firing rate at threshold can be expressed using the following equation:

1 $$v_{\mathrm{f}} = v_0 + a\left[ {{\mathrm{ePT}}} \right]_ +$$

where *v*_0_ is the initial build-up rate (either $v_0^{\mathrm{L}}$ or $v_0^{\mathrm{R}}$), *a* is the acceleration due to perceptual information (either *a*_T_ or *a*_D_), ePT is the effective processing time, equal to rPT minus afferent and efferent delays, [*x*]_+_ = max{0, *x*} (i.e., negative ePT values were rounded to zero), and *v*_f_ is the derivative of the firing rate at threshold. Thus, the two terms in Eq. (1) represent urgency-driven (internally-derived and not based on the cue information; *v*_0_) and perceptual (cue-derived; *a*[ePT]_+_) influences on the ongoing oculomotor activity. To illustrate the differential contributions of urgency and perceptual information to our observed results, we simulated thousands of trials of the model and plotted the mean *v*_0_ and *a*[ePT]_+_ terms separately as functions of rPT (Fig. 7h, i). At short rPTs, as expected, perisaccadic motor plans were void of any perceptually-based signal and were driven solely by urgency. However, interestingly, at long rPTs, urgency and perceptual information modulated perisaccadic motor plans in opposite directions, on average. That is, for long-rPT correct choices, the mean initial build-up rate (*v*_0_) decreased, while the influence of the acceleration due to perceptual information at threshold crossing (*a*[ePT]_+_) increased with processing time. For long-rPT incorrect choices the opposite was true; i.e., *v*_0_ increased slightly on average, while *a*[ePT]_+_ decreased on average with increasing processing time. Therefore, after ~125 ms of cue viewing time, saccade peak velocity, inasmuch as it relates to the state of perisaccadic oculomotor plans, carries a multiplexed signal, which, as evidenced by our simulations, is predominantly driven by perceptual information.

### Covert and overt correlates of urgent-decision confidence

**Fig. 8:**
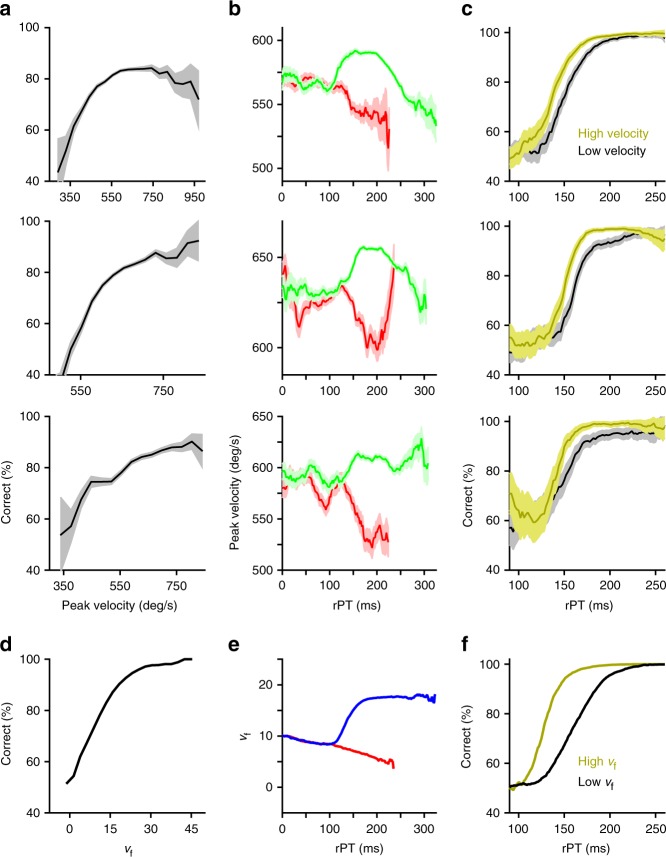
Saccade peak velocity and simulated oculomotor neural data correlate with the probability that a choice is correct, given the perceptual evidence. **a** Percentage of correct responses as a function of saccade peak velocity for each of the three subjects. **b** Mean (±1 s.e.m.) peak velocity of correct (green) and incorrect (red) saccadic choices as a function of rPT (previously shown in Fig. 3a). **c** Percentage of correct responses as a function of rPT for trials with peak velocities above (gold) and below (black) the median peak velocity. Error bars in **c** represent 95% binomial proportion confidence intervals. **d** Percentage of correct responses as a function of the derivative of simulated neural activity at threshold crossing (*v*_f_). **e** Mean *v*_f_ of correct (blue) and incorrect (red) simulated trials as a function of rPT. **f** Percentage of correct simulated trials as a function of rPT for trials with high (above median) and low (below median) *v*_f_. Data are from monkeys R, T, and G (rows 1–3). Simulations (row 4) were based on the behavioral data from subject R. For all simulated results, a small amount of noise was added to *v*_f_

But, what is the significance of this top-down mental computation to the saccadic choice itself? Our results thus far indicate that, in the CS task, saccade peak velocity and *v*_f_ reflect the degree to which perceptual evidence is weighted for or against the target of the saccadic choice around the time of commitment. Based on this intuition, we hypothesized that, in the CS task, saccade peak velocity and *v*_f_ might closely correlate with the probability that a choice is correct given the perceptual evidence — i.e., with the statistical definition of decision confidence^24–26^. The key insight here is that, in the urgent-decision paradigm, processing time itself quantifies target/distracter discriminability, because it directly determines the amount or strength of the perceptual evidence that is available in each trial. Equating processing time with discriminability, we found that, in the CS task, saccade peak velocity exhibits three analytically derived signatures of statistical decision confidence^24^ (although see ref. ^27^ for limitations regarding the generality of such signatures). First, for all three subjects, choice accuracy increased as a function of saccade peak velocity (Fig. 8a). Second, as previously shown, the average peak velocity of correct trials increased, while that of incorrect trials decreased as a function of rPT (Fig. 8b). And third, saccadic choices with higher peak velocities were associated with enhanced perceptual performance as a function of rPT when compared to those with lower peak velocities (Fig. 8c). As hypothesized, running the same analyses on the perisaccadic firing rate derivatives (*v*_f_) obtained from simulations of the accelerated race-to-threshold model yielded similar results. In plotting the simulated curves, we found that adding a modest amount of noise to *v*_f_ served to more closely replicate the peak velocity data (Fig. 8d–f; see Methods). The only discrepancy is that the simulated performance curves conditioned on *v*_f_ showed a slightly exaggerated relative shift, which was likely due to the absence of downstream sources of noise in the model, i.e., noise beyond the motor planning stage (e.g., FEF), which would inevitably influence the kinematic/confidence signal as it descends from cortex. These data indicate that, in the CS task, the peak velocities of saccadic choices overtly manifest unsolicited, covert measures of decision confidence, which, according to our predictions, are computed within oculomotor circuitry simultaneously with the choice around the time of its execution.

## Discussion

We investigated, on a fine temporal scale, whether and how perceptual decision-making dynamics influence the metrics of saccadic choices upon execution. Our results revealed that various saccade metrics (e.g., peak velocity, amplitude, vigor, endpoint scatter) are highly dependent on processing time (rPT) — the amount of time available to evaluate sensory information that is relevant for guiding a motor choice. This dependence was characteristically similar across saccade metrics, manifested both in measures of their mean and variability, and followed a similar time course (both in onset and rate) to that of choice accuracy. Through our simulations, we provided a physiologically plausible mechanistic explanation of our behavioral results, demonstrating how perceptually-driven changes in neural activity within oculomotor structures (e.g., FEF) may influence saccade peak velocity. In addition, we discovered that, in the CS task, peak velocity and simulated FEF data (*v*_f_) exhibit multiple features that are characteristic of the statistical definition of confidence^24^ (although confidence results have been shown to take other forms^27,28^).

While our results do suggest that, consistent with previous findings, saccade metrics are modulated by urgency-based signals^29^, the effects observed in this study were predominantly based on perception. The difference between rPT and RT is critical. Indeed, by design, systematic changes in urgency are minimized in the CS task — avoiding the fundamental relationship between urgency and response time that is characteristic of RT, and affording a clearer view of the impact of perceptual information on saccadic choice behavior. By analyzing many thousands of CS task trials, we were able to detect, as a function of rPT, a small (e.g., see Fig. 2c) yet highly robust influence of perception on saccade metrics — an effect otherwise easily missed.

Previous work has established that the metrics of a saccade are influenced by the prior history of reward associated with the location of space to which it is made^30–35^. However, in the CS task, the outcome of each trial is in no way contingent upon that of previous trials, and thus, our main findings cannot be directly attributed to systematic alterations of either motivational or decision-related variables across extended periods of time (for related effects, see refs.^5,22^). Rather, our results indicate that saccade metrics are modulated by rapid changes in cognitive state that occur within a single trial, unfold over a few tens of milliseconds, and are based primarily on incoming sensory evidence.

Still, our results are likely related to the subject’s internal valuation of the saccade target at the time of choice commitment. Indeed, our data indicate that, under urgent conditions, saccade metrics are influenced by the perceptual evaluation process that is inherent to covert visual target selection in general. Viewed in this way, our results are broadly in agreement with and expand upon those of studies that have shown saccade metrics to be modulated by the reward or value associated with the target of a saccadic eye movement^30–39^. As we discuss further below, much can be inferred about (1) the dynamics of the perceptual decision-making process, (2) the neural mechanisms by which sensory evidence influences saccadic choice kinematics, and (3) the computations associated with the covert selection and overt execution of rapid perceptually-guided saccadic choices, based on the current findings.

Our results indicate that, under urgent circumstances, covert, graded measures of visual evidence are not entirely lost in the sensorimotor transformation upon the selection of a discrete, binary choice, but rather are largely preserved and communicated through the metrics of saccades upon execution. That is, in the CS task, saccade metrics appear to reflect the degree to which sensory evidence is weighted for or against the target of the saccadic choice around the time of commitment. Accordingly, as a function of processing time, saccade metrics revealed the dynamics of the underlying perceptual judgment — manifesting intimate and otherwise hidden details of the perceptual evaluation process as it unfolded in time. For example, that incorrect saccades were slower, shorter, and had less reliable endpoints with increasing rPT indicates that our subjects had, to a certain extent, accurately perceived the cue information, despite their failure to indicate as much via their binary saccadic choice selections (additional analyses of corrective saccade metrics further corroborate this idea; Supplementary Fig. 2). Such clues about the perisaccadic state of perceptual processing go well beyond what can be inferred based on standard psychophysical measures such as RT or choice accuracy alone.

Our behavioral findings strongly implicate oculomotor brain regions in a sensorimotor transformation whereby perceptually-driven changes in activity influence saccade kinematics. To gain insight into the neural mechanisms by which this could occur, we utilized the accelerated race-to-threshold model, which has previously been shown to replicate both CS-task performance metrics (e.g., choice accuracy, rPT distributions, RT distributions) and simultaneously recorded FEF neuronal data^5,6,21,22^. Using the model, fit to RT data and blind to all saccade metrics (e.g., peak velocity, endpoint scatter, etc.), we demonstrated how perceptually-driven changes in neural activity within oculomotor structures could influence saccade peak velocity — based on the speed with which a motor plan crosses threshold. These simulations help bridge the gap between studies that have found perceptual modulation of FEF motor neuron activity^5,6^ and those that have, through microstimulation^17,18^, or inactivation^40–45^, demonstrated that FEF activity influences saccade peak velocity. In contrast, however, the amplitude and direction (and, by association, endpoint) of saccadic eye movements are primarily encoded by the locus of neural activity within FEF and SC oculomotor maps^10–14^, and thus, inferences regarding these metrics are beyond the scope of the accelerated race-to-threshold model. With further experimental validation, this newly proposed physiological link between perception and saccadic choice kinematics could become an important constraint on neural models of oculomotor decision making in general.

Extant data from subcortical structures are not inconsistent with the proposed link based on the model results. While uncertain of the exact ensemble of brain regions that, in concert with the FEF, may instantiate the apparent influence of the perceptual evaluation process on saccade peak velocity, previous work implicates the caudate nucleus as well as the SC, both of which receive direct projections from the FEF^46–49^. Indeed, not only have putative perceptual decision variables been shown to be encoded within the firing rates of caudate and SC neurons^3,4,50,51^, but activity within these brain regions has also been demonstrated to correlate with saccade peak velocity^15,16,30,52^.

We discovered what appear to be unsolicited measures of decision confidence embedded within the kinematics of eye movements in the CS task. Equating saccade peak velocity with decision confidence, our results indicate that, during a simple color discrimination, sensory evidence starts to inform the computation of decision confidence only ~125 ms after cue onset, with confidence reaching its full extent of modulation only ~60 ms after that. Moreover, we can infer, based on the results of Fig. 3b, that sensory evidence informs the computation of confidence with the same onset time and rate of change with which it informs rapid saccadic choices. Together, these behavioral results suggest that confidence is computed within oculomotor circuitry along with the choice — and, possibly, as an inherent part of the urgent decision-making process itself. This conclusion is in agreement with the results of recent experiments carried out in the lateral intraparietal area^53^. Here, we provide further support for this idea with our neural simulations, which predict that, in the CS task, statistical decision confidence is encoded within FEF motor neuronal activity that is causal to the choice.

Our behavioral data are in agreement with the predictions of normative as well as signal detection-theory based models of confidence^24,54^, and our simulations seemingly represent a natural extension of such models to the context of urgent perceptual decision making. The emergence of this latent computation — statistical decision confidence — within the model and within saccade metrics gives further credence to the accelerated race-to-threshold framework, and highlights the utility of saccade metrics as a behavioral medium through which covert perceptual decision-making dynamics can be inferred. Evidently, although largely stereotyped, saccades are highly communicative, thoughtful movements, which under time pressure can provide basic insight into the neural computations that give rise to perceptually-guided choice behavior.

## Methods

### Subjects and setup

Three male rhesus monkeys (*Macaca mulatta*) participated in the experiment. All experimental procedures were conducted in accordance with NIH guidelines, USDA regulations, and the policies set forth by the Institutional Animal Care and Use Committee (IACUC) of Wake Forest School of Medicine. Each animal was implanted with an MRI-compatible titanium post under general anesthesia. The post served to fix the head in place during experimental sessions.

Eye movements were recorded using an EyeLink 1000 infrared tracking system (SR Research), operating in pupil-corneal reflection mode, with a sampling rate of 500 Hz. Visual stimulus generation, task sequencing, and eye movement data acquisition were accomplished via a custom-designed PC-based software package (Ryklin Software). Stimuli were presented on a display monitor at a viewing distance of 57 cm.

### Behavioral task

Details of the CS task have been described previously^5,6,21,22^. Briefly, on a given trial of the CS task (Fig. 1a), the subject fixates on a centrally located spot, the color (red or green) of which defines the color of the eventual target. While the subject fixates, two identical yellow spots appear, diametrically opposed, surrounding the central stimulus. The yellow spots serve as placeholders, informing the subject of the two potential locations of the correct target. Then, the fixation spot disappears, representing the command to move (the go signal). However, at this time, the identities of the target and distracter are unknown and will remain so for a variable gap of time (25–250 ms), until the “cue” period begins. At cue onset, one yellow spot turns green and the other red. The subject’s choice is indicated via a saccadic eye movement made at any point after the go signal. Two important quantities are measured: reaction time (RT) and rPT. The RT is measured as the time that elapses between the onset of the go signal and the onset of the saccadic response. The rPT is the maximum cue viewing time; it is measured as the time between cue onset and saccade onset (rPT = RT − gap). Subjects were required to fixate within a criterion window (3° radius) around the correct target for a specified duration (typically, 200 ms) to receive a drop of juice. Trials in which the correct target was red were randomly interleaved with trials in which the correct target was green. Negative rPT responses correspond to those executed after the go signal, yet prior to cue onset (i.e., during the gap). For this rare subset of trials, the correct target was randomly assigned to be on the left or right despite there being no cue information provided, and thus, subjects had a 50% chance of being correct.

Monkeys were first trained to perform a non-urgent version of the two-alternative forced choice task, in which the color cue is presented before the go signal, to learn the decision rule of matching the color of a peripheral spot to that of the fixation point. Following proficient performance of the non-urgent two-choice task (i.e., performance > 95% correct), subjects started performing the CS task, which, as described above, instantiates a variable gap of time between the presentation of the go signal and the cue. To encourage short latency response times, subjects had to respond within approximately 450 ms following the go signal (i.e., with a RT < 450 ms); otherwise, the trial timed out. Monkeys quickly adapted to this time constraint (indeed, they prefer not to wait^21^) and typically mastered the CS task within a matter of days to a few short weeks with no further instruction.

### Data analysis

All analyses were performed in Matlab (MathWorks, Natick MA). For behavioral data analysis, only trials with targets presented at 10° of visual angle directly to the left and right of screen center were analyzed. The *X* and *Y* positions of the eye, represented in degrees of visual angle relative to the center of the screen, were smoothed using a Gaussian kernel with a standard deviation of 1 sample. Smoothed *X* and *Y* eye position data were then transformed into radial coordinates and differentiated to calculate eye velocity in the radial direction. Similar results were obtained with different smoothing kernels, kernel widths, as well as with no smoothing at all. In addition, no qualitative differences were observed using vectorial velocity in place of radial velocity. The start and end times/positions of a saccade were defined according to the sample indices at which eye velocity exceeded or fell below 25°/sec. All saccades less than 6° of visual angle or greater than 13° of visual angle in amplitude were excluded from analysis. Saccades with peak velocities greater than 1000°/sec were excluded as well. In total, less than 2% of saccades were excluded.

Average and s.d. of metrics as functions of rPT were computed with bin widths between 25–50 ms, and with step sizes of 1–2 ms. Percentages of correct responses were calculated as functions of peak velocity (as well as *v*_f_) using bin sizes equal to one tenth the range of peak velocities (and *v*_f_ values) and with step sizes equal to one half the bin size.

The following procedure was used to calculate saccade endpoint scatter. First, for each session, we calculated the distance from each saccade endpoint to the mean endpoint of all saccades toward the corresponding choice stimulus. The resulting distance measures from each session were then converted to *z*-scores, and, across all sessions, averages were computed in rPT bins for correct and incorrect trials separately. The resulting curves, plotted in Fig. 6a, represent the mean displacement in saccade endpoint relative to the mean endpoint of saccades, as a function of rPT.

Similar results were obtained when the displacement of each saccade endpoint was measured relative to the mean endpoint of rPT- and outcome-matched saccades (rather than relative to saccades across all rPTs regardless of outcome as described above). This analysis provided confirmation that the endpoint effects observed are due, in large part, to changes in the spread of the distributions of endpoints as a function of rPT, rather than simply resulting from shifts in the means of the distributions of endpoints as a function of rPT.

The peak velocities of saccades toward each choice stimulus were first z-scored within each session before taking rPT-binned standard deviation measures as shown in Fig. 6b. As explained in the Results section, to determine whether the observed mean peak velocity effects were beyond those predicted by the saccadic main sequence, we computed the rpv around the line of best fit between the amplitudes and peak velocities of uninformed saccades (rPTs < 75 ms). We used a linear rather than an exponential fit here because the trials analyzed had fixed potential target locations, and thus the ranges for saccade amplitude and peak velocity were quite small.

Curves representing yoked (correct and incorrect) modulation in peak velocity as a function of rPT (such as those seen in Fig. 3b) were generated by subtracting incorrect from correct peak velocity averages within rPT bins. The same procedure was used to generate a single modulation curve for *v*_f_, which was then compared (as described below) to that of mean peak velocity. Similarly, for saccade endpoint scatter, curves were generated by subtracting correct from incorrect endpoint scatter averages within rPT bins. These curves were used to compare the time course of modulation of endpoint scatter to that of choice accuracy, with respect to rPT.

Curve rise times (for tachometric curves, peak velocity modulation curves, etc.) were calculated by fitting the data of interest with a piece-wise-linear version of a sigmoid function, such that

$$f\left( x \right) \,\, = \,\,\left\{ \hfill \begin{array}{*{20}{l}} {y_1} \hfill & {{\mathrm{if}}\,\,\,x \leq x_1} \hfill \\ {y_1} + \left( {x - x_1} \right) \ast \left( {\frac{{y_2 - y_1}}{{x_2 - x_1}}} \right) \hfill & {{\mathrm{if}}\,\,\,x_1 < x < x_2} \hfill \\ {y_2} \hfill & {{\mathrm{if}} \,\,\, x \geq x_2} \hfill \end{array} \right.$$

The set of parameters (*x*_1_, *x*_2_, *y*_1_, *y*_2_) that achieved the overall minimum sum of squared residuals between the linear sigmoid function and the data was found by a combination of analytical and numerical methods. The rise time of the resultant best-fitting linear sigmoid was then measured as:

$${\mathrm{rise}}\,{\mathrm{time}} = x_2 - x_1$$

To determine the temporal alignment between two curves (e.g., one representing changes in peak velocity and the other changes in choice accuracy, such as those plotted together in Fig. 3b), we varied the baseline, rescaled the *y*-axis, and shifted the *x*-axis of one curve until the absolute difference between the two curves was minimized. The resulting *x*-shift value of the minimization solution was our measure of temporal shift (relative alignment) between the two curves.

Bootstrapping procedures^55,56^ were used to estimate the degree to which two modulation curves followed the same time course. That is, the data were resampled with replacement and the metrics of interest (e.g., difference between the rise times of two curves, or temporal shift between curves, as defined above) recomputed thousands of times, generating a distribution of values based on the data. From each bootstrapped distribution, a 95% confidence interval was computed. Zero fell within the 95% confidence interval for each tested measure, indicating that there were no significant differences between the time courses of the modulation curves compared.

Next, we estimated the probability that the rise time (as well as the position along the time axis) of one modulation curve was either greater (later) or smaller (earlier) than that of another curve just by chance. To do so, a distribution of differences was obtained based on the bootstrapped distributions of the individual curves (as described above), and from it, the probability of obtaining a value more extreme than zero was calculated (two-tailed).

### Model simulations

Accelerated race-to-threshold model simulations were carried out with procedures nearly identical to those described in previous reports^6,22^. After an afferent delay (drawn randomly for each trial from a Gaussian distribution) following the presentation of the go signal, two variables (*x*_L_, *x*_R_), which represent the mean firing rates of two populations of oculomotor neurons, begin racing toward a fixed threshold (1000 units) at constant build-up rates ($v_0^{\mathrm{L}}$, $v_0^{\mathrm{R}}$, drawn randomly for each trial from a bivariate Gaussian distribution with a negative correlation coefficient). Trials in which *x*_L_ or *x*_R_ reach threshold during this stage (i.e., before the cue influences simulated motor plans) represent guesses and result in simulated choices that have 50% chance of being correct. Otherwise, after a second afferent delay (drawn randomly for each trial from a Gaussian distribution) following the presentation of the cue, both motor plans pause for a brief period of time (fixed across trials). Then the motor plan congruent with the correct target location accelerates (*a*_T_; fixed across trials), while the motor plan congruent with the distracter location decelerates (*a*_D_; fixed across trials) until either the race is over (i.e., one of the plans reaches threshold), or, a maximum or minimum velocity is reached (*v*_max_, *v*_min_). When either *x*_L_ or *x*_R_ reach threshold, a saccade is assumed to occur after a short efferent delay (fixed across trials), allowing for simulated performance measures to then be computed (trial outcome, RT, rPT, etc.). Overall, the model has 12 free parameters, including the means, variances, and, when applicable, the correlation coefficients of the aforementioned Gaussian distributions from which values are drawn on each trial. These 12 parameters were optimized for each subject during the model fitting procedure (see Model Fitting section below).

For the simulations shown in Fig. 8, Gaussian noise was added to all *v*_f _values, under the constraint that *v*_f_ values could not fall below zero. The amount of noise was manually adjusted until the similarities between the simulated and peak velocity data appeared optimal based on visual comparison. This procedure was found to generate results that more closely replicated the peak velocity data.

Given that, in the accelerated race-to-threshold model framework, changes in the slope of the firing rate are predominantly attributed to perceptual influence, the *v*_max_ and *v*_min_ parameters primarily represent the maximum rates at which sensory evidence can inform (i.e., enhance or suppress) saccadic motor planning. Although these two parameters are exactly as in published versions of the model, they (and their possible variability) play somewhat unique roles in linking the present model results to the saccade metrics data, particularly at very long rPTs. For all trials, the upper limit on the slope of the firing rates at threshold crossing (*v*_f_) is equal to *v*_max_, but the implicit lower limit on *v*_f_ is zero because the firing rate cannot reach threshold with a negative slope. This explains why *v*_f_ (and peak velocity) plateaus for correct but not for incorrect trials. During correct trials, as rPT increases, the probability that the slope of a simulated motor plan congruent with the target will equal *v*_max _increases, eventually reaching 100%, and so does the probability that the motor plan will reach threshold to trigger a correct choice. Both quantities reach their limits. In contrast, during incorrect trials, the plan that is incongruent with the target decelerates, but the lower its slope, the less likely it is to reach threshold in the first place, to trigger an error. Thus, in this case, *v*_f_ (and peak velocity) does not typically reach its implicit lower limit, and so it does not flatline.

With only slight adjustments to these parameters, the model can easily account for additional subtle features observed in the saccade metrics data. For instance, although in the current instantiation of the model *v*_max_ is fixed across trials, adding variability to it results in *v*_f_ decreasing, on average, for correct trials after ~225 ms of processing time (not shown), replicating the decreases in the mean peak velocity of correct saccadic choices observed at very long rPTs for monkeys R and T (Figs. 3a, 5c). This would suggest that the eventual decreases in peak velocity of correct choices simply reflect trial-to-trial variability in the maximum rate at which sensory evidence informs a saccadic choice. However, many other interpretations and mechanistic explanations of this behavior are possible, some of which are related to post-decisional processes. Thus, we decided to focus primarily on the effects that occur during the most relevant decision-related time frame (i.e., rPTs < 200 ms) which can be explained by the preexisting accelerated race-to-threshold model framework without adding parameters to the model as published previously.

### Model fitting

The free parameters of the model were optimized such as to minimize the mean absolute error between the simulated and monkey reaction time distributions within each gap, separately for correct and incorrect trials, as done in previous studies of the CS task^5,6,22^. Correct and incorrect trial distributions within each gap were normalized relative to the same value during the fitting procedure, ensuring that the relative frequency of correct versus error trials was preserved and thus that each distribution was weighted appropriately. Crucially, we did not have multiple, unique sets of model parameters dedicated to simulating trials with a given gap length, or trial outcome (correct/incorrect). Rather, one set of model parameters was used across all trials within a simulated session, no matter the gap or trial outcome. The best-fitting set of parameter values was found by exhaustive search; i.e., by generating many sets of parameter values (each parameter being drawn randomly from its own distribution) and selecting the set of parameter values that, upon running the model, minimized the aforementioned error. This procedure was then repeated, narrowing the width and shifting the mean of each parameter distribution toward parameter values that yielded better fits, following each block of search. All simulations shown were from a model that was fit to the RT data from subject R (our largest behavioral dataset presented here, with 19,796 trials).

We stress that saccade metrics did not enter into the model or fitting procedure in any way. Rather, after fitting the model to the behavioral data, the model was run and the simulated responses were used to compute *v*_f_ in each trial.

### Data availability

All relevant data and code used for the analysis are available from the authors upon reasonable request.

## Electronic supplementary material

Supplemental Information
