# Supplementary material for: Saccade metrics reflect decision-making dynamics during urgent choices
Source: Nat Commun. 2018 Jul 25;9:2907. doi: 10.1038/s41467-018-05319-w (PMC6060154; doi:10.1038/s41467-018-05319-w)
Supplement: Supplementary file 1 — Supplemental Information [file 41467_2018_5319_MOESM1_ESM.pdf]

Supplementary Information

Saccade metrics reflect decision-making dynamics during urgent choices

Seideman et al.

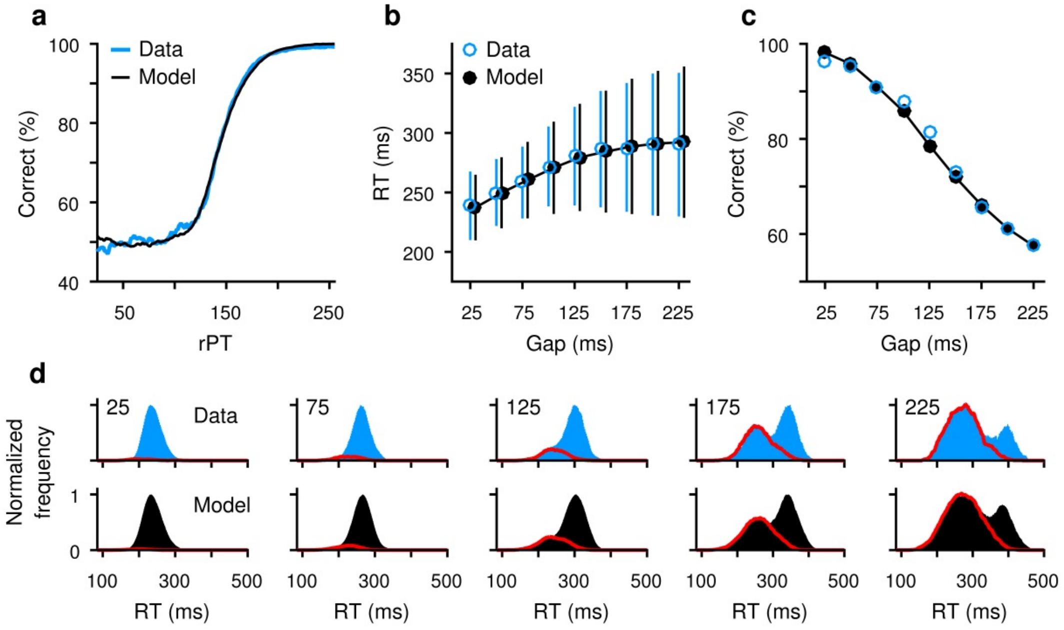

Supplementary Figure 1. Results of the accelerated race-to-threshold model fitted to the behavioral data. Simulations in figures 7 and 8 were generated with the same set of model parameters fit to the reaction time and choice data shown here. No saccade metrics (e.g., peak velocity) data were available to the model at any point during the fitting procedure. **a**, Percentage of correct responses as a function of rPT (tachometric curve). **b**, Mean RT (± 1 s.d.) as a function of gap duration (chronometric curve). Data points include both correct and incorrect trials. **c**, Percentage of correct responses as a function of gap duration (psychometric curve). Trials with longer gaps typically result in shorter rPT responses, and thus lower accuracy (and vice versa). **d**, Reaction time distributions at five gaps for correct (blue and black bars) and incorrect (red lines) trials. Gap duration is indicated in the upper left corner of each column. For all panels, results labeled 'Data' are from Subject R; results labeled 'Model' are from simulations of the accelerated race-to-threshold model.

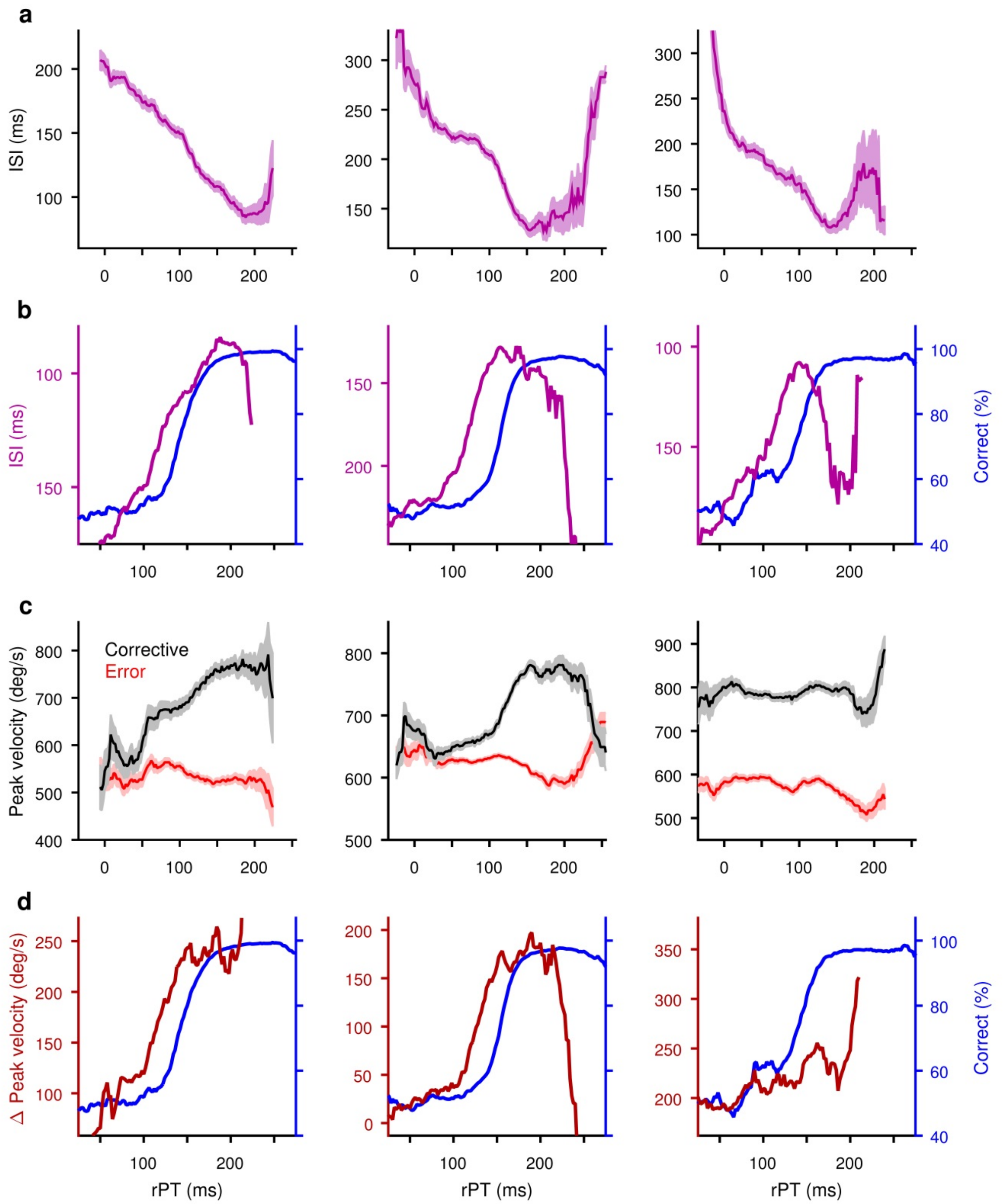

Supplementary Figure 2. Cue viewing time prior to an incorrect choice modulates corrective saccade metrics. **a**, Mean intersaccadic interval (ISI; ± 1 s.e.m.) between the end of the first incorrect response to the distracter and the onset of the second corrective saccade executed to the target as a function of rPT. **b**, ISI (inverted and rescaled along the y-axis; purple) and choice accuracy (blue) as functions of rPT. **c**, Mean peak velocity (± 1 s.e.m.) of incorrect (red) and corrective (black) saccades as functions of rPT. **d**, Mean difference in peak velocity between each first (incorrect) and second (corrective) saccade (dark red) as a function of rPT. Choice accuracy (blue) as a function of rPT. Data are from monkeys R, T, and G (left to right). For all monkeys, the longer the processing time, the shorter the ISI. In two of the three monkeys, the peak velocity of a corrective saccade was strongly modulated by the amount of processing time on which the previous, erroneous saccade was based.
